# Control of Spring Softening and Hardening in the Squared Daisy

**DOI:** 10.3390/mi12040448

**Published:** 2021-04-16

**Authors:** Mathieu Gratuze, Abdul-Hafiz Alameh, Seyedfakhreddin Nabavi, Frederic Nabki

**Affiliations:** 1 Department of Electrical Engineering, École de Technologie Supérieure, Université du Québec, Montréal, QC H3C 1K3, Canada; mathieu.gratuze.1@ens.etsmlt.ca (M.G.); abdul-hafiz.alameh.1@ens.etsmtl.ca (A.-H.A.); snabavi@mun.ca (S.N.); 2 Department of Electrical and Computer Engineering, McGill University, Montréal, QC H3A 0G4, Canada

**Keywords:** resonant behavior characterization, duffing resonators, hardening, softening, non-linearity, micro-resonator, piezoelectric transducer, frequency response

## Abstract

Nonlinear, mechanical microelectromechanical system (MEMS) resonating structures exhibit large displacement and a relatively broad operating bandwidth. These unique features make them particularly of interest for the development of MEMS actuators and sensors. In this work, a mechanical MEMS structure allowing the designer to determine the type of nonlinearity, that is, softening or hardening, based on its anchor scheme is presented. Effects of the excitation signal on the behavior of the proposed MEMS in the frequency domain are investigated. In this regard, a comprehensive experimental comparison among the nonlinear behaviors of softening and hardening has been conducted. To reduce the hysteresis effect to a minimum, an excitation approach, which is a pulsed sweep in frequency with a discrete resolution, is presented. The maximal velocity, quality factor, bandwidth, and resonant frequency of these two types of nonlinear MEMS resonators are compared under three different types of excitation. Finally, it is shown that the performance and characteristics extracted from nonlinear mechanical MEMS resonating structures are highly dependent on the excitation method. Hence, in the present case, the apparent performances of the MEMS resonator can increase by up to 150% or decrease by up to 21%, depending on the excitation approaches. This implies the necessity of a standardized testing methodology for nonlinear MEMS resonators for given end applications.

## 1. Introduction

Nonlinearities of microelectromechanical system (MEMS) resonators have been recognized as a limitation to their normal operation. As a result, the presence of nonlinear behavior in MEMS resonators can compromise their performance. As such, several techniques and resonator architectures have been proposed to reduce or compensate for the influence of nonlinearity [1,2,3]. On the other hand, nonlinear behavior is usually desired in vibration insulators [4,5] and vibration energy harvesters (VEH), as they can leverage a larger displacement and a broader bandwidth [6,7,8]. Such relatively large displacement is also highly desirable for the conception of active time differentiators for terahertz applications [9,10]. Moreover, nonlinear resonators have been the subject of recent interest for the design and development of nonlinear MEMS accelerometers, resonator switches, and logic gates [11,12,13,14].

To exert nonlinear phenomena in MEMS devices, specific design guidelines are required. The expression of such nonlinear stiffness phenomena can be regrouped into two categories: spring softening and spring hardening. In the first case, increasing of the amplitude of the excitation will lead to a reduction of the resonant frequency, while in the second case, this increase of the amplitude of the excitation will lead to an augmentation of the resonant frequency. In terms of practical application of the nonlinear phenomenon in MEMS devices, there does not appear to be a preference for either type of nonlinearity for specific applications, as softening- and hardening-type VEH, resonators, and switches have been presented in the literature [15,16,17]. However, it can be expected that in the future, as more and more of these devices are designed, preferences for targeted applications will appear. This highlights the necessity of developing clear guidelines and structures, allowing the designers to choose either spring softening or hardening of the MEMS devices.

Characterization of nonlinear MEMS performance is important to verify the accuracy of the simulations and determine the actual properties of the devices. However, such nonlinear MEMS devices typically exhibit strong hysteresis phenomena. Such hysteresis renders the characterization more complex, as it causes the performance of the MEMS devices to depend on the previous state. Characterization methods that reduce the impact of hysteresis in nonlinear resonators have been developed. Among them, “FREEVIB” presented in [18,19] and “FORCEVIB” presented in [19,20] are the most prominent. However, such characterization methods are time-consuming, complex to implement, sensitive to noise, and generally reserved for macro-sized structures. For these reasons, alternatives to such methods have been presented [21,22]. The application of such a method to nonlinear MEMS resonators as presented in [23], and remains the exception. Accordingly, characterization methods that reduce the impact of hysteresis are not widely employed in the literature touching on the design and development of nonlinear MEMS resonators, as can be observed in [6,7,8,11,12,13,14,15,16,17,24,25,26,27]. Therefore, developing experimental characterization methods that allow for mitigation, or at least relative control of such hysteresis in nonlinear MEMS resonators is desirable.

In the literature, several architectures that favor the nonlinear behavior in MEMS resonators have been proposed. However, few of these studies investigate both the softening and hardening responses of the same design. Accordingly, the aim of this work is to study the impacts of different anchoring schemes on the frequency response of piezoelectric MEMS nonlinear resonators, and develop a characterization method allowing for relative control of the hysteresis. In this regard, by using two different topologies, the presence of hardening and softening behaviors in the nonlinear MEMS resonator are studied. The findings of this study highlight the necessity of controlling the characterization of nonlinear MEMS resonators as a function of the end application. In such a system, the characterization approach greatly impacts the performance of the MEMS devices in terms of efficiency and operational frequency range. Thus, the contributions of this work can be listed as follows:

1. A MEMS resonator architecture allowing the designer to readily control the type of nonlinearity, that is, yielding either spring hardening or softening;
2. An experimental testing methodology allowing the monitoring and control of the hysteresis in the nonlinear resonators; and
3. Recommendations for the characterization of nonlinear resonators.

The remaining parts of this paper are structured as follows: a general background on how to induce the nonlinearity in the MEMS resonators is presented in Section 2. The design and fabrication process of the studied structures is summarized in Section 3. In Section 4, the performance of nonlinear MEMS resonators as a function of the excitation signal is discussed, and the influence of the hysteresis is shown. Section 5 provides a comprehensive comparison of the practical implementation of such changes in the performance of MEMS resonators. Finally, a conclusion is presented.

## 2. Materials and Methods

### Control of the Nonlinearity in MEMS Structures

During the design phase, the MEMS designer can exploit three main types of effects to induce nonlinearity in the MEMS device: *damping*, *forcing*, and *stiffness* [28]. Some *damping* effects have been shown to be inherently nonlinear, such as squeeze film damping [29]. In the case of the *forcing* effect, it can be exploited to induce nonlinearity by using an external force (e.g., capillary attraction, Van der Waals forces, electrostatic actuation, magnetic forces). Some of these effects have been successfully demonstrated in MEMS devices [30,31]. Finally, changes in the stiffness of the MEMS structure will lead to nonlinearity, and these changes of stiffness can be attained by using certain materials, particularly piezoelectric ones [28]. These changes in stiffness can also be induced by the use of geometrical nonlinearity, as MEMS devices generally undergo relatively large deformation [32]. This geometrical nonlinearity can be caused by a wide range of factors, such as large deflections or rotations, initial stresses, or load stiffening. These effects are particularly noticeable in the stretching of thin structures.

**Figure 1:**
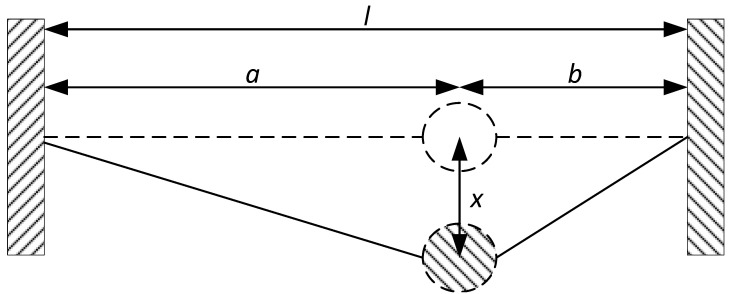
Illustration of the weighted string mechanical system, Reprinted with permission from ref. [33]. Copyright 2010 McGraw-Hill Handbooks.

Nonlinear resonant geometries have been of particular interest for both physicists and mechanical engineers. In the case of a MEMS designer, the use of such previously characterized geometric nonlinearity will allow for the control of the nonlinear behavior of the MEMS device. Among these structures, some are particularly interesting, since they can naturally exhibit either a softening or a hardening response. The weighted string is such a system. Depending on its initial parameters, it is capable of exhibiting either spring softening or spring hardening, or even a linear response [33]. This system is comprised of string of a half-length $l_{0}$ with a weight of a mass *m* located at distance *a* from one anchor and *b* from the other anchor. *x* denotes the displacement of the mass. Such a system is shown in Figure 1. To analytically describe this behavior, an approximation of the governing nonlinear differential equation is given by [33]:

(1) $$m\ddot{x}+F_{0}\left(\frac{1}{ab}\right)x+\left(SE-F_{0}\right)\left(\frac{a^{3}+b^{3}}{2a^{3}b^{3}}\right)x^{3}=0,$$

where $F_{0}$ is the initial tension, *S* is the cross-sectional area of the string, and *E* is the elastic modulus of the string. In this system for the specific case where a=b, the value of κ, the amplitude-frequency coefficient, can be expressed as:

(2) $$\kappa =\frac{SE-F_{0}}{4a*F_{0}}.$$

In such a case, the initial tension is equal to [33]:

(3) $$F_{0}=SE\left(\frac{a-l_{0}}{l_{0}}\right).$$

Therefore, by substituting (3) in (2), (2) can be rewritten as:

(4) $$\kappa =\frac{2*l_{0}-a}{4a*(a-l_{0})}.$$

According to this equation, depending on the initial values of *a* and $l_{0}$ different spring behaviors (i.e., linear, softening, or hardening) can be observed. Therefore, the MEMS designer can leverage such a mechanism by carefully choosing the design values to have a nonlinear structure leveraging either softening or hardening behavior.

It should be noted that the weighted string is inherently nonlinear, as the anchoring of the mass is made using strings that can be in tension, but cannot bend. At the MEMS scale, such strings cannot be readily implemented and must be replaced by beams (which can have various shapes) that have a different underlying dynamic.

However, the idea of using a central proof mass anchored on both sides to induce nonlinearity in the MEMS structure has been successfully employed in the literature to design nonlinear resonators with either spring softening or spring hardening characteristics. In [8], a resonator was designed to have softening behavior, while in [6,24], a resonator with spring-hardening behavior is presented. A similar but more complex structure, such as the one presented by [7] exhibits spring-hardening behavior, where three proof masses were used. However, to the best knowledge of the authors, no such micro-structure based on the idea of a central proof mass and designed to exhibit *either* spring softening *or* hardening behavior has been presented in the literature.

It should be noted that the weighted string is not the only architecture that allows for the designer to control the type of nonlinearity exhibited by the system. As such, [25] has shown that the coupling of 45 ${}^{\circ }$ inclined springs will result in either spring hardening, softening, and even linear behavior. However, the focus of that work is more on the analytical modelling of such nonlinearity. Work presented in [26] showed that the coupling of two cantilevers with a multi-walled boron nitride nanotube (BNNT) can result in either spring hardening or spring softening. However, this method requires post-processing of the MEMS and has only been demonstrated at the nanoscale.

## 3. Design and Micro-Fabrication Process

### 3.1. Design

**Figure 2:**
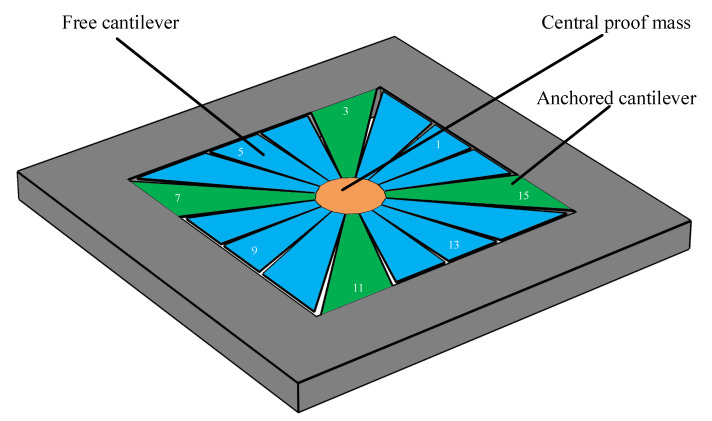
Illustration of one of the available anchoring schemes of the squared daisy MEMS resonator. In this anchoring scheme, the cantilevers 3, 7, 11, and 15 are anchored and shown in green, while the others are free and shown in blue.

As was previously discussed, MEMS architectures with nonlinear behavior have been proposed in the literature. However, few allow for the control of their nonlinear behavior to support *either* spring softening *or* hardening behavior. In this paper, one of these architectures, the squared daisy (SD) structure, is studied w.r.t. its nonlinear behavior. This architecture was presented for the realization of a MEMS piezoelectric VEH in [27]. The SD structure can be partially simplified to the weighted string structure, as it consists of a central proof mass acting as the weight suspended by a predefined number of cantilevers of varying cross-sections, as shown in Figure 2. These cantilevers can either be anchored or free. By choosing which of the cantilevers will be anchored and act as the string, and the ones that are only clamped on the central proof mass, the MEMS designer can carefully determine the spring-mass parameters of the structure. In Figure 2, the free cantilevers are illustrated in blue, while the anchored ones (i.e., supports) are shown in green. The central mass is shown in an orange color.

A basic analytical analysis of the resonant frequency in the SD structure was presented in [27], where it was shown that the fundamental resonant frequency of such a structure can be expressed as [27]:

(5) $$f_{0}=\left(\frac{1}{2\pi }\right)*\sqrt{\frac{48EI}{\left(2\pi R_{m}\rho H\right)*\left(L-\left(2R_{m}\right)S_{c}\right)}},$$

where *E* is the Young’s modulus of the material, *I* is the area moment of inertia, which depends on the physical dimensions of the device, $R_{m}$ is the radius of the proof mass, *H* is the height of the proof mass, ρ is the density of the material, *L* is the length of the structure, and $S_{c}$ denotes the scaling factor.

However, it should be noted that these equations can only be used for an approximate estimation of the resonant frequency, since the cross-sectional area in the proposed MEMS nonlinear resonator is not uniform over the entire cantilever length. Furthermore, this analytical model does not take into account the nonlinear behavior of the system.

In terms of behavior, the displacement of central mass is expected to dictate the global behavior of the system. If this displacement is small, then the spring constant of the anchoring cantilevers can be assumed as linear, and therefore the global behavior of the structure will be linear. However, for larger displacements, the supporting cantilevers will be deflected and stretched, eventually exhibiting non-linear behavior. This implies that the spring constant of the SD will vary with the magnitude of the displacement. To predict the behavior of the supporting cantilevers, it is necessary to either make an analytical model or use FEM simulations.

Therefore, to validate and accurately predict the behavior of the SD structure under different anchoring schemes, simulations have been performed using the COMSOL Multiphysics software package, version 5.5, COMSOL Inc., Stockholm, Sweden. These simulations are aimed to extract the mode shape, resonant frequency, and spring constant under varying loads when the anchoring scheme is changed.

**Figure 3:**
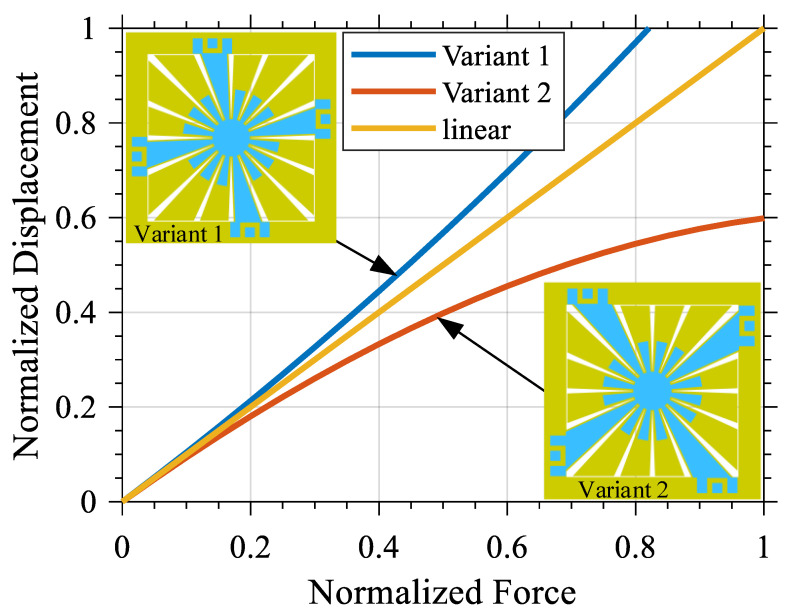
Influence of the force applied on the deflection of the central proof mass for both SD resonator variants.

As a result of this process, two SD variants have been defined. The parameters used to describe these variants are presented in Table 1. These parameters have been carefully chosen to simulate MEMS devices fabricated using the PiezoMUMPs micro-fabrication process. The results of the simulation of the behavior of such variants, under different loads, are shown in Figure 3. Consequently, it has been shown that the SD structure has the potential to exhibit either softening or hardening behavior depending on its anchoring scheme, since such an anchoring scheme effectively changes the behavior of spring in the presence of a large-enough displacement.

The thicknesses and geometry of the different layers have been chosen to match the guidelines of the PiezoMUMPs micro-fabrication process from MEMSCAP, Crolles, France. The selected anchoring schemes are presented in Table 1 and shown in Figure 3. It should be noted that due to the rotational symmetry in the SD structure, several configurations result in the same anchoring scheme. Simulation and experimental measurements of the mode shape of each defined variant is presented in Appendix A.

### 3.2. Fabrication

**Figure 4:**
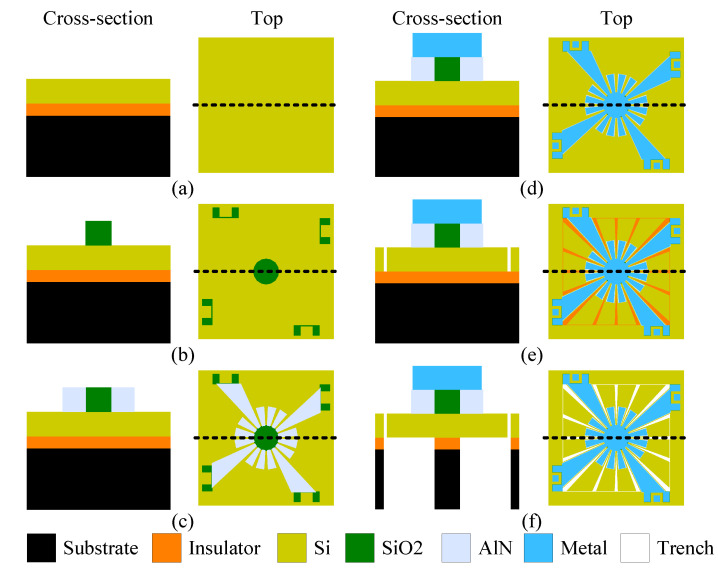
Simplified overview of the PiezoMUMPs’ fabrication process flow, applied to the fabrication of Variant 2 of the Squared Daisy.

To experimentally validate and study the effects of nonlinearity on the SD structure, the variants previously presented were fabricated. In this section, the PiezoMUMPs’ micro-fabrication process to prototype the MEMS resonators described in Section 3.1 is described. This process provides cost-effective access to piezoelectric MEMS prototyping. In the literature, this process has been reported for the implementation of various MEMS-based resonators and VEH (e.g., [7,27,34,35,36,37]). The fabrication process includes five masks based on an N-type, double-sided, polished silicon-on-insulator (SOI) wafer. In the first step, a 10 μm-thick silicon (Si) layer in the (001) orientation (Figure 4a) is doped in order to increase its electrical conductivity for use as a bottom electrode. Thereafter, an insulating 0.2 μm-thick layer of silicon dioxide is grown and patterned on the SOI wafer (Figure 4b). A 0.5 μm-thick piezoelectric layer of aluminum nitride (AlN) is then deposited and patterned (Figure 4c). In the next step, a layer of metal is deposited, consisting of a stack of 20 nm-thick chromium (Cr) and of 1 μm-thick aluminum (Al). This layer is used as the top electrode (Figure 4d). The silicon device layer is then patterned to create the suspended structure (Figure 4e). Then, the 400 μm substrate is etched from the backside of the wafer to form the trench below the structure to release it (Figure 4f). It is worth noting that this process allows for the use of the suspended substrate to be used as a proof mass. Accordingly, in the case of the SD structure, the trench step also frees the proof mass to enable it to vibrate. Upon reception of the device from the foundry, no post-processing step has to be applied to the manufactured devices. Complementary information regarding the fabrication process can be found in [38].

**Figure 5:**
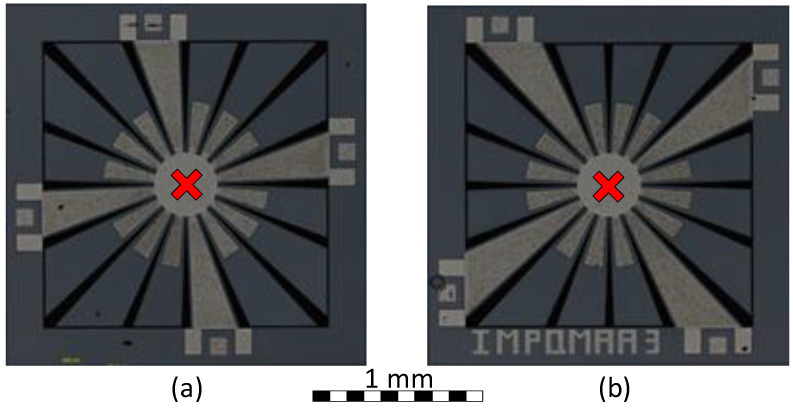
Micrograph of the fabricated SD devices: (**a**) Variant 1 (hardening) and (**b**) Variant 2 (softening). The different anchoring schemes for each structure can be identified, along with an indication of the vibration measurement point used.

The layouts for both SD resonator variants as defined in Table 1 were implemented. The fabricated resonators occupy a total silicon area of 1700 by 1700 μm, and are shown in Figure 5. In this figure, the proposed anchoring schemes and the point used for the vibration measurement are shown.

## 4. Experimental Results

### 4.1. Description of the Experimental Test Setup

To characterize the hysteresis and nonlinearity behaviors of the MEMS resonators described in Section 3, the following approach was carried out. The prototyped SD resonators were electrically excited, while their mechanical responses were measured by an optical vibrometer. It is worth mentioning that in the case of MEMS piezoelectric transducers, a mechanical or an electrical excitation will yield similar behavior in frequency. In the present case, an electrical excitation was used, since a simpler measurement setup is required for that purpose.

**Figure 6:**
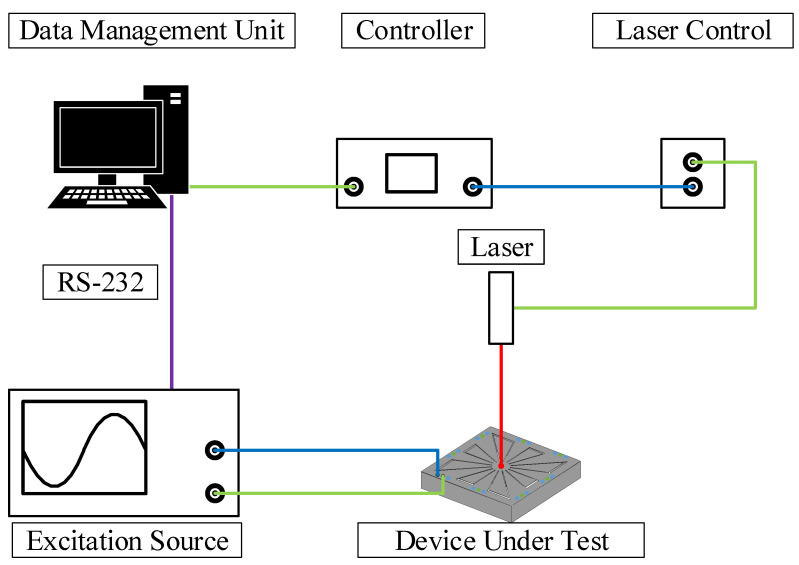
Schematic of the vibrometer test bench.

The vibrometer used for the experimental test setup was acquired from Polytec, Irvine, CA, USA, and includes a data management system, a vibrometer controller (OFV-2570), and a laser unit (OFV-534). This test setup is presented in Figure 6. In order to satisfy the Nyquist-Shannon sampling theorem, the sampling frequency $F_{s}$ for all the measurements was set to be 25.6 kHz. To excite the prototyped MEMS resonators, a function generator type 33250A from Keysight, Santa Rosa, CA, USA was used. This function generator was controlled by the serial interface to provide the desired voltage excitation signal. As marked in Figure 5, the deflection of the central mass was considered as the measurement point. The deflection measurements of the free cantilevers will yield a similar response with a higher velocity due to the increased degree of freedom of such cantilevers, as shown in [27]. This can also be directly observed using the measurement of the mode shape of both variants presented in Appendix A.

### 4.2. Description of the Excitation Signals

The MEMS resonators were excited by using three different excitation voltage signals—namely, pulsed sweep (PS), where the excitation frequency is discretely swept, continuous sweep forward (CSF), where the excitation frequency was swept in an ascending manner, and continuous sweep backward (CSB), where the excitation frequency was swept in a descending manner. The nature of these excitation voltage signals are described in further detail below.

In the nonlinear regime, the hysteresis is dependent on the prior state of the vibrating system. To eliminate the hysteresis effect in the characterization of the MEMS resonator, a pulsed sweep PS was defined. When excited with a PS-type excitation, the MEMS devices are excited at a particular frequency $F_{i}$, for a given duration of $T_{on}$; afterwards, the devices are turned off for a duration of $T_{off}$. The value of $F_{i}$ is sequentially swept between $F_{start}$ and $F_{end}$, the lower and the higher excitation frequencies, respectively. The excitation frequencies are thus discrete, and the resolution of this PS is equal to the distance between two consecutive excitation frequencies, $F_{res}$.

The PS has been carried out following three main variations. In the first one, the excitation frequencies’ steps are applied in an increasing order. Then in the second, the excitation frequencies’ steps are applied in a decreasing order. Finally, in the third, the excitation frequencies’ steps are applied in a random order. These sweeps have been named pulsed sweep forward (PSF), pulsed sweep backward (PSB), and pulsed sweep random (PSR), respectively. By performing these three pulsed sweeps, the test setup can be validated, and the hysteresis effect of the characterization can be eliminated (i.e., by using a sufficient $T_{off}$ duration to eliminate the hysteresis in the system). The minimal duration of $T_{off}$ varies with each resonator, as it is a function of the quality factor *Q* and the resonant frequency of the resonator. Both of these parameters are responsible for the decay time of the resonator τ. Experimental results have shown that if $T_{off}$ is greater than 10 τ, then the hysteresis effect of the characterization is effectively eliminated. It should be noted that the resolution in frequency for this characterization mode is equal to $F_{res}$, and is affected by neither the sampling frequency nor the duration of the excitation (provided that the Nyquist-Shannon sampling theorem is respected).

**Figure 7:**
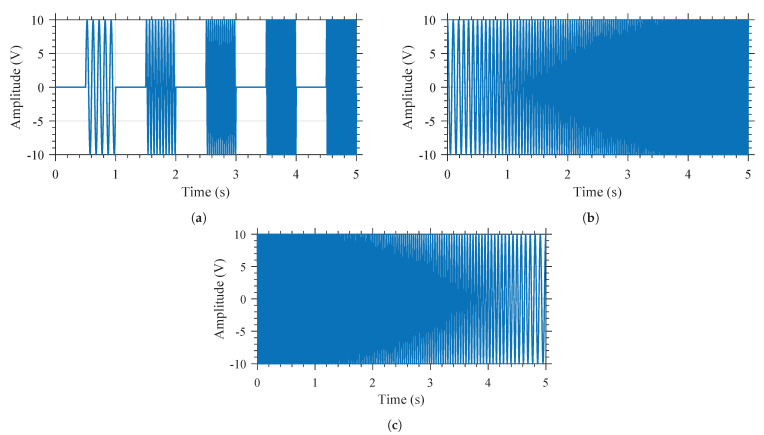
Examples of three of the excitation signal types used: (**a**) *PSF*, (**b**) *CSF*, and (**c**) *CSB*.

An illustration of the excitation signal for the (PSF) is shown in Figure 7a. In this case, the amplitude of the excitation signal is set to be 20 V, while $F_{start}$ and $F_{end}$ are 10 Hz and 50 Hz, respectively. $F_{res}$ is 10 Hz. The duration of $T_{on}$ and $T_{off}$ are identical, that is, 0.5 s. The overall excitation is carried out over a duration of $T_{s}=5s$.

In order to exert the hysteresis behavior of the devices, two continuous sweep excitation signals were used, where the frequency was swept continuously in an ascending manner, named continuous sweep forward (CSF), and in a descending manner, named continuous sweep backward (CSB). For these excitation signal sweeps, the start and end frequencies of the sweep ($F_{start}$ and $F_{end}$), as well as the duration of the excitation $T_{e}$ are set. These parameters result in a sweep rate $S_{r}$ and a resolution in frequency $F_{res}$. $S_{r}$ can be expressed as:

(6) $$S_{r}=\left|\frac{F_{end}-F_{start}}{T_{e}}\right|.$$

However, experimental results have shown that varying the value of $S_{r}$ from 8 Hz/s up to 6250 Hz/s does not have an influence on the behavior in frequency of the resonators. On the other hand, $F_{res}$ is entirely dependent on the sampling frequency $F_{s}$ and the number of points considered for the FFT operation $N_{FFT}$, and can be expressed as:

(7) $$F_{res}=\frac{F_{s}}{N_{FFT}}.$$

As the value $N_{FFT}$ is a function of the duration of the excitation, reducing the value of $T_{e}$ will increase the value of $F_{res}$, and therefore reduce the ability to accurately characterize the behavior of the nonlinear MEMS resonator. This effect can be partially compensated by using zero-padding on the measured signal, as this operation will artificially augment the number of points considered for the FFT operation.

An illustration of the excitation signal for the CSF and CSB is shown in Figure 7b,c, respectively. With reference to this figure, the amplitude of the excitation signal is 20 V. The lower and higher frequencies are 10 Hz and 50 Hz, respectively. The excitation duration is $T_{e}=5s$.

It is worth reminding that the term of “frequency response” and “resonant frequency” are somehow inaccurate for the nonlinear resonators, as the hysteresis effect comes into action. Hence more accurately described, the behavior in frequency of the resonators in presented, as caused by a defined stimulus. In the case of nonlinear resonators, contrary to linear resonators, the resonant frequency varies as a function of the amplitude of the excitation signal and the type of excitation provided to the resonator (i.e., while for a linear resonator knowing the amplitude and frequency of the excitation is enough to determine the displacement of the resonator, in the case of nonlinear resonators, the previous state should also be specified to allow such determination). Hence, the term “resonant frequency” will not be used, and instead the term frequency at which the maximum velocity is reached ($F_{mv}$) will be used. This effect will be demonstrated in the following sections, where the responses of the devices over frequency to different excitation amplitude levels and to the excitation signal types described above will be shown.

### 4.3. Signal Parameters for Each of the Excitation Signals

To allow for a comparison of the continuous sweeps forward and backward (CSF and CSB) signals, the sweep parameters have been carefully chosen to allow a relative comparison between each of them. These parameters are presented in Table 2. These parameters have been chosen to provide a wideband excitation signal but also allow for relative comparison between the two excitation schemes, as the value of $T_{e}$, $S_{r}$, and $F_{res}$ are kept constant to 500 s, 8 Hz/s, and 2 mHz, respectively.

In the case of the PS excitation, the parameters of the signal for both devices are presented in Table 3. These parameters have been chosen to provide a large band excitation signal, and also to allow a relative comparison between the (CSF and CSB) excitation signals.

For these three types of excitation, (PS, CSF, and CSB), measurements will be performed for different signal amplitudes, namely, 5 V, 10 V, 15 V, and 20 V.

### 4.4. Summary of the Measurement Results

**Figure 8:**
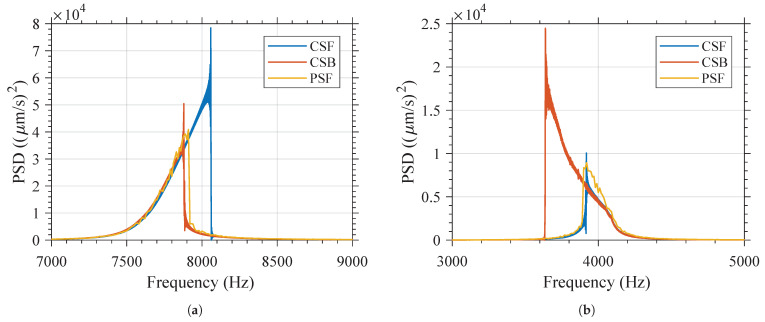
Influence of the excitation signal type (CSF, CSB, or PSF) on the frequency behavior when the amplitude of the excitation signal is 20V (**a**) Variant 1 and (**b**) Variant 2.

The measurement results in the time and frequency domains when modifying the amplitude of the excitation signal for both variants can be found in Appendix B for the CSF- and CSB-type excitation. In the case of the PS-type excitation, the measurement results in the frequency domain when modifying the amplitude of the excitation signal and the order of the excitation of the frequencies for both variants can be found in Appendix C. Figure 8 presents a summary of the measurement results for each variant under CSF-, CSB-, and PSF-type excitation. In this figure, the amplitude of the excitation voltage is 20 V. The influence of the type of excitation on the behavior in frequency of the resonator can be clearly seen.

In Table 4, the measurement results are summarized. The influence on the amplitude of the excitation voltage and the type of excitation signal on the maximal velocity reached, the frequency at which the maximum velocity is reached ($F_{mv}$), and bandwidth of both variants are presented. The measurement results using the pulsed sweep (PS)-type excitation have been summarized in the columns PS, as PSF-, PSB-, and PSR-type excitation yield similar results. In this table, the bandwidth has been defined as the full width at half maximum (FHWM) (i.e., the frequency range in which the amplitude is equal or greater than 50% of the maximal velocity reached).

**Figure 9:**
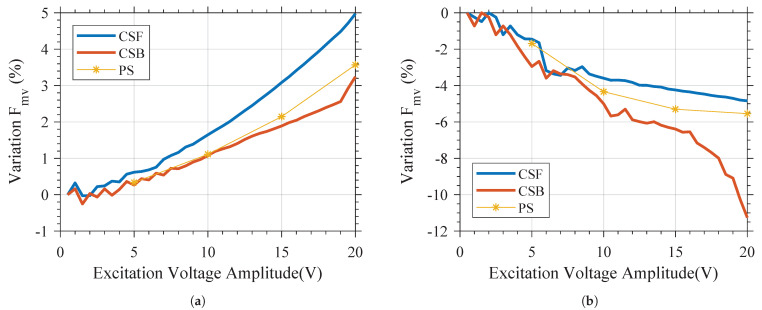
Influence of the amplitude and type of the excitation (*CSF*, *CSB*, or *PSF*) on Fmv of (**a**) Variant 1 and (**b**) Variant 2.

Contrary to the behavior of a linear resonator, for which the variation of the amplitude and type of the excitation signal does not result in variation of the resonant frequency, it can be seen that the behavior of both the variants in the frequency domain is highly dependent on the amplitude and type of the excitation signal. For all excitation types, increasing the amplitude of the excitation signal results in a higher $F_{mv}$ for Variant 1, but in a lower $F_{mv}$ for Variant 2. As shown in Figure 9, if the amplitude of the excitation voltage is constant, it can be observed that the reduction of $F_{mv}$ for Variant 2 is greater than the increase of the $F_{mv}$ of Variant 1. This is in line with the simulation results shown in Figure 3. As shown in that figure, at a constant force, the difference between the deflection of Variant 2 and linear behavior is greater than the difference between the deflection of Variant 1 and linear behavior.

It can be concluded that Variant 1 exhibits a hardening-type spring softening, while Variant 2 exhibits a softening-type spring softening. It is also shown that if the amplitude of the excitation signal is too low (below ≈ 3 V), the amplitude of the excitation voltage does not have a strong influence on $F_{mv}$, and both devices appear to behave linearly as the displacement of the central proof mass is too small to induce the geometrical nonlinearity.

The frequency behavior of the device can be modified according to the excitation technique. As for Variant 1, as shown in Figure 8a, it can be clearly seen that when the SD device is subjected to the CSF excitation signal, it has a larger displacement amplitude in comparison to the CSB excitation signal; while on the contrary for Variant 2, as shown in Figure 8b, it can be seen that when the SD device is subjected to the CSB excitation signal, it has a larger displacement amplitude in comparison to the CSF excitation signal.

It is also interesting to note from Figure 8 that the characterization using the pulsed sweep (PS)-type excitation (i.e., PSF, PSB, or PSR) will have similar behavior to the characterization using CSB-type excitation, but a slightly higher maximal velocity and $F_{mv}$ for Variant 1. A similar observation can be made regarding Variant 2 and CSF-type excitation. This can also be seen in Figure 9, where the variation in the $F_{mv}$ is plotted versus the excitation voltage, showing that as the amplitude is increased, the CSF-type excitation for Variant 1 and the CSB-type excitation for Variant 2 yield a resonant frequency that diverges significantly from that obtained using PS excitation. These variations are not linked to the resolution in frequency of the PS signal.

Consequently, as it has been simulated and measured, the SD structure has the potential to exhibit different nonlinear behavior depending on the selected anchoring scheme. Moreover, it has been shown that using a PS-type excitation can allow for relative control of the hysteresis in nonlinear resonators.

## 5. Discussion

Nonlinear resonators are being considered for the realization of VEH, resonators, switches, and logic gates. Depending on the end application, the excitation of the nonlinear resonators will vary. If the nonlinear resonators are employed as actuators or sensors, they will be electrically driven at a defined frequency, such that the behavior in frequency of these nonlinear resonators will be closer to the results obtained with the PS-type excitation.

If the nonlinear resonators are destined for energy-harvesting applications, they will be subjected to the vibrations in the ambient. Due to their nature, mechanical vibrations cannot be predicted, and therefore, the characterization of VEH using a method that will give different results depending on the previous state of the system will lead to inaccurate results.

Therefore, pulse swept signals have been chosen as the reference, as in the case of a PS excitation, the hysteresis effect is removed from the characterization. To compare the effect of such excitation signals on the characterization of the same resonator (Variant 1 or 2), the amplitude of the excitation signal was set to 20 V, and the three different signals were applied: (PSF, CSF, and CSB). For the pulsed sweep, for simplicity, only the PSF results are presented, as it has been previously demonstrated that it will yield a similar response to the other PS methods (PSB or PSR). The resulting maximal velocity, $F_{mv}$, bandwidth, quality factor, and resonance amplitude are presented in Table 5 for both variants. As explained previously, the reference has been set to the performances of the devices when excited with a PSF-type excitation.

As such in the present case, characterizing a device presenting spring softening behavior with a CSF instead of a PS-type excitation will lead to a global underestimation of its characteristics, as the maximal velocity, $F_{mv}$, and bandwidth will be underestimated by 1.31%, 0.05%, and 13.64%, respectively. This will lead to an overestimation of the quality factor of 16.67%. On the contrary, characterizing a device presenting spring softening behavior with a CSB instead of a PS-type excitation will lead to a general overestimation of its characteristics, as the maximal velocity and bandwidth will be overestimated by 86.81%, and 33.64%, respectfully, as well as an underestimation of both $F_{mv}$ and of the quality factor by 7.19% and 33.33%, respectively.

On the other hand, characterizing a device presenting spring-hardening behavior with a CSF instead of a PS-type excitation will lead to a global overestimation of its characteristics, as the maximal velocity, $F_{mv}$, and bandwidth will be overestimated by 23.28%, 1.87%, and 10%, respectively. This will lead to an underestimation of the quality factor of 7.14%. Characterizing a device presenting spring-hardening behavior with a CSB instead of a PS-type excitation will lead to a global underestimation of its characteristics, as the maximal velocity, $F_{mv}$, and bandwidth will be underestimated by 0.81%, 0.39%, and 21.07%, respectively. This will lead to an overestimation of the quality factor of 28.57%.

In the case of energy harvesters, this overestimation is particularly of interest, as the maximum output power is directly linked to the maximum displacement, and therefore, the velocity reached by the resonator, and the operation range of the resonator is linked to the bandwidth of the resonance. Therefore, a figure of merit (FOM) has been defined as the product of the amplitude of the velocity at resonance by the bandwidth of the resonance. This FOM is therefore expressed in Hzmm/s. This FOM allows for the realization of a compromise between the bandwidth and amplitude of the displacement at resonance, both weighted equally. Such FOM is presented in Table 5 for both variants. This shows that the FOM can be skewed significantly depending on how the devices are characterized.

Similarly to what has been observed previously, characterizing a device presenting with nonlinear behavior with a CSF-type excitation will lead to an underestimation (−15% in the present case) or an overestimation (36% in the present case) of its characteristics, depending on whether the device is presenting with spring softening- or spring hardening-type behavior.

On the other hand, characterizing a device presenting with nonlinear behavior with a CSB excitation will lead to an overestimation (150% in the present case) or an underestimation (−22% in the present case) of its characteristics, depending on whether the device is presenting with spring softening- or hardening-type behavior.

Therefore, the authors recommend the characterization of nonlinear MEMS resonators to be performed using a PS-type excitation. However, the characterization of a nonlinear resonator with such excitation is a bit more complex to implement than the CSF and CSB excitations. As function generators are not readily suited to such an operation, it is necessary to remotely control the excitation frequency, using either a remote interface capability or arbitrary signal generation. A manual measurement of the frequency behavior, frequency by frequency, to carry out a PS excitation can also be carried out if test time is not a factor. In this work, automated test sequences were programmed using the signal generator interface to reduce the test time.

It should be noted that if PS-type excitation is not available to the designer, the type of excitation used should be specified to allow the reader to estimate whether the characteristics presented are either overestimated or underestimated. Exceptions to this recommendation can be made if the excitation signal of the nonlinear resonators is known to exhibit a sweep in frequency while in use in a given end-application, in which case the characterization using a CSF or a CSB-type excitation will lead to more accurate results depending on the type of the frequency sweep expected in the application.

## 6. Conclusions

In this paper, the characterization and control of the nonlinearity in MEMS resonators were studied. This was done by investigating the effects of the anchoring scheme on the nonlinearity of the squared daisy structure (SD). Two variants of this structure were simulated, fabricated, and measured. Although all the designs occupy the same area, their nonlinear behavior in the frequency domain is not identical due to their anchoring scheme. One anchoring scheme results in spring softening, while the other results in spring hardening, thus allowing the designer to tune the behavior of the nonlinear MEMS. It was also shown that the excitation scheme used to characterize the resonators is important, as it can affect the characterization of the device, and lead to different extracted performance metrics. Accordingly, the use of pulsed sweeps (PS) to characterize the behavior of these nonlinear MEMS resonators was presented. PS excitation allows for an accurate characterization of nonlinear MEMS resonators, as it allows the removal of the impact of hysteresis on the frequency behavior of nonlinear resonators. The authors recommend the use of PS-type excitation as the standardized testing methodology for nonlinear MEMS resonators for most end-applications.

Ultimately, the recommendations stemming from this work can help provide a better understanding of the considerations for the design and characterization of nonlinear MEMS resonators, and to ensure that their characterization is done with excitation signals that are appropriate.

## Figures and Tables

**Table 1:** Overview of the parameters for each of the SD resonator design variants

	Variant 1	Variant 2
Size of the design (μm × μm)	1700 × 1700	
Radius of the proof mass, Rm (μm)	200	
Thickness of the substrate (μm)	400	
Thickness of the cantilevers (μm)	10	
Cantilever used as anchors	1 5 9 13	3 7 11 15
Free Cantilever	2 3 4 6 7 8 10	1 2 4 5 6 8 9
	11 12 14 15 16	10 12 13 14 16
Resonant frequency (simulation)	8100	4050
Nonlinear behavior (simulation)	Hardening	Softening

**Table 2:** Characteristics of the CS-type excitation signal

	Excitation Type	$F_{\mathrm{start}}$ (kHz)	$F_{\mathrm{end}}$ (kHz)	$T_{e}$ (s)
Variant 1	CSF	6	10	500
	CSB	10	6	
Variant 2	CSF	2	6	500
	CSB	6	2	

**Table 3:** Characteristics of the PS excitation signal

	$F_{\mathrm{start}}$ (kHz)	$F_{\mathrm{end}}$ (kHz)	$F_{\mathrm{res}}$ (Hz)	$T_{\mathrm{on}}$ (s)	$T_{\mathrm{off}}$ (s)
Variant 1	6	10	10	1.06	0.53
Variant 2	2	6			

**Table 4:** Summary of the characteristics of each variant for different excitation signal types

	Excitation Voltage (V)	Variant 1			Variant 2		
		*PS*	*CSF*	*CSB*	*PS*	*CSF*	*CSB*
Maximal Velocity (mm/s)	5	90.0	93.3	93.2	20.7	17.0	42.5
	10	176.6	181.50	181.7	95.0	95.7	118.0
	15	234.0	261.7	234.7	109.6	110.1	193.3
	20	271.1	334.2	268.9	122.1	120.5	228.1
$F_{mv}$ (Hz)	5	7750	7722	7716	4080	4197	4002
	10	7800	7812	7797	3970	3969	3854
	15	7860	7929	7839	3930	3942	3675
	20	7910	8058	7879	3920	3918	3638
Bandwidth (Hz)	5	180	185	182	150	213	87
	10	180	163	169	160	196	188
	15	230	234	193	180	156	265
	20	280	308	221	220	190	294
Quality factor	5	43	42	42	27	20	46
	10	43	48	46	25	41	21
	15	34	34	41	22	25	14
	20	28	26	36	18	21	12

**Table 5:** Summary of the performances of Variants 1 and 2 under different excitation when the amplitude of the excitation signal is of 20 V

	Excitation Type	Variant 1	Variant 2
Overestimation of the velocity (%)	CSF	−1.31	23.28
	CSB	86.81	−0.81
Overestimation of $F_{mv}$ (%)	CSF	0.05	1.87
	CSB	−7.19	−0.39
Overestimation of the bandwidth (%)	CSF	−13.64	10.00
	CSB	33.64	−21.07
Overestimation of the quality factor (%)	CSF	16.67	−7.14
	CSB	−33.33	28.57
FOM ($\mathrm{Hz}mm\;s^{-1}$)	PSF	26,862	75,908
	CSF	22,895	102,934
	CSB	67,061	59,427
Overestimation of the FOM (%)	CSF	35.60	−14.77
	CSB	−21.71	149.64

## Appendix A. Presentation of the Mode Shape of the Resonators

The vibrometer test setup presented in Figure 6 has been used to experimentally measure the mode shape of each variant. To make such acquisition, a grid made of 2500 linearly spaced points has been defined. The position of each point of this 50 × 50 grid has been carefully defined using a positioning controller (Corvus Evo from PI) and 2 positioners (VT-80 Linear Stage from Physik Instrumente (PI), Karlsruhe, Germany).

The experimental data has been acquired using the CSF-type excitation for variant 1 and the CSB-type excitation for variant 2 as described in Table 2. The amplitude of the excitation has been set to 20 V. In order to reduce the time needed to perform the whole experiment, the excitation duration has been set $T_{e}=640ms$. This in turns results in $S_{r}=6250\mathrm{Hz}/s$.

After acquisition of the data, the movement of the structure can be reconstructed at a defined frequency, as the exact coordinates where the points are measured are known. The experimental measurement of the mode shapes of both proposed variants are presented in Table A1. This table also presents the simulated mode shapes obtained using the COMSOL Multiphysics software package. The good agreement between the simulated and experimentally measured mode shape can be seen.

In each figure it is possible to identify the displacement of the free and anchored cantilevers. The data has been is scaled relatively to the largest displacement present in the mode shape, which means that for the Variant 2 some free cantilever almost appear as not moving while in fact they are, but amplitude of the displacement of those cantilevers is lower than other free cantilever in the structure.

**Table A1:** Visualisation of the mode shape of the structure

	Mode Shape	
	Simulation	Measurement
Variant 1		
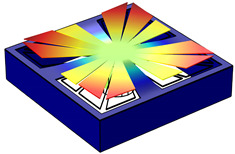
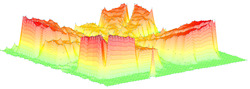
Variant 2		
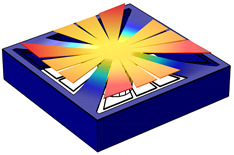
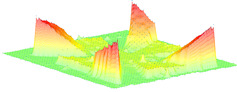

## Appendix B. Excitation with Continuous Sweep Signals

In Table A2, the response of variants 1 and 2 in the time domain is shown along with the frequency content of such signal when the devices are excited with a CSF or CSB-type excitation. For clarity, the frequency range shown in this figure has been limited to the interval between 7 kHz and 9 kHz for the variant 1 and between 3 kHz and 5 kHz for the variant 2.

The influence of the excitation amplitude on the resonant frequency of the variants is clearly noticeable. For variant 1, the greater the amplitude, the greater the resonant frequency. Such an increase is greater for CSF excitation. For variant 2, the greater the amplitude, the lower the resonant frequency. Such a decrease is greater for CSB excitation.

Such results validate the simulation results which predicted a hardening type response for variant 1 and softening type response for variant 2. This also shows the dependency of the frequency domain behavior of nonlinear MEMS resonators in response to the type of excitation used.

It should be noted that sharp peaks just before the down-jump or up-jump phenomena are shown in the frequency domain, these sharp peaks are artifacts due to the signal processing as it can be seen that these peaks do not exist in the time domain.

**Table A2:** Summary of the measurements results for the CS-type excitation

Excitation Type	Measurements in the:	
	Time Domain	Frequency Domain
Variant 1 CSF		
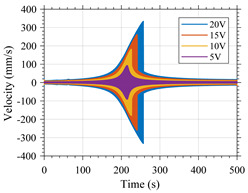
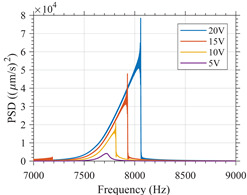
Variant 1 CSB		
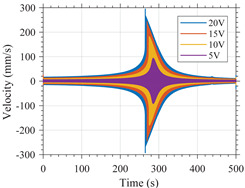
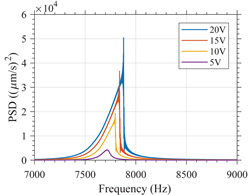
Variant 2 CSF		
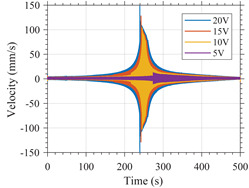
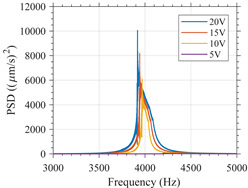
Variant 2 CSB		
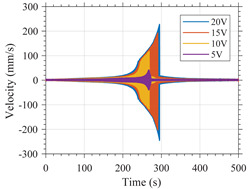
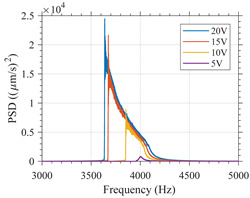

## Appendix C. Excitation with Pulsed Sweep Signals

In Table A3, the behavior over frequency of both variants when varying the amplitude of the excitation signal is shown. For clarity, the frequency range shown in this figure has been limited to the interval between 7 kHz and 9 kHz for the variant 1 and between 3 kHz and 5 kHz for the variant 2. The influence of the order in which the frequencies are applied when the devices are excited with a PS-type excitation is also shown in the table. As can be seen, the order in which the frequencies are applied has no significant impact on the behavior in frequency of both variants, as the results for the PSF, PSB and PSR are identical. Therefore, the characterization of the nonlinear resonators using a PS-type excitation allows for the characterization of the nonlinear resonator devices without the effect of hysteresis.

Such results validate the simulation results which predicted a hardening type response for variant 1 and softening type response for variant 2. This also validates that it is possible to mitigate the hysteresis behavior in nonlinear MEMS resonators by characterizing them using a PS-type excitation.

**Table A3:** Summary of the measurements results for the PS-type excitation

	Influence of the:	
	Amplitude of the Excitation Signal	Order of Excitation of the Frequency
Variant 1		
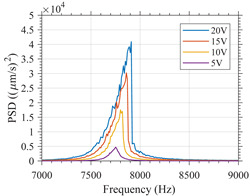
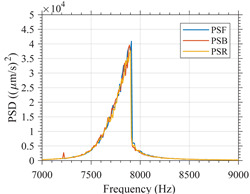
Variant 2		
